# Survival of patients with cancer associated thrombosis at the Uganda Cancer Institute

**DOI:** 10.3332/ecancer.2021.1212

**Published:** 2021-03-25

**Authors:** Clement D Okello, Yusuf Mulumba, Abrahams Omoding, Henry Ddungu, Jackson Orem

**Affiliations:** Uganda Cancer Institute, Upper Mulago Hill Road, P.O. Box 3935, Kampala, Uganda

**Keywords:** thrombosis, cancer, Uganda

## Abstract

**Background:**

The occurrence of venous thromboembolism (VTE) in patients with cancer leads to a reduced life expectancy. There is an increased incidence of cancer and its associated mortality in Uganda. We described the survival and characteristics of patients with cancer associated thrombosis (CAT) in a tertiary oncology centre in Uganda.

**Methods:**

We performed a retrospective study on patients with CAT at the Uganda Cancer Institute (UCI) using a homogenous purposive sampling method.

**Results:**

One hundred and eleven patients with documented VTE were included in the analysis. At entry, the mean age was 52.4 years, and 69 were female. Ninety eight had deep venous thrombosis, while 12 had pulmonary embolism. The most common cancer diagnoses were haematologic (30), gynaecologic (20) and prostate (17) cancers. Treatment regimens included anticoagulation with low-molecular weight heparin (LMWH) (72) and combined LMWH with warfarin (22). The median overall survival (OS) was 6.3 months, with a 1-year survival rate of 41.5%. Patients with significantly increased hazard of mortality were those with upper gastrointestinal (UGI) malignancies, colorectal and breast cancers. Patients with a body mass index of 25–29.9 kg/m^2^ (overweight) had a slightly reduced hazard of mortality.

**Conclusion:**

The OS of patients with CAT at the UCI is short. Most patients with CAT presented with advanced stage cancers and at a relatively young age. Patients with UGI, colorectal and breast cancers had increased hazards of mortality, whereas those who were overweight had a slight reduction in the hazard of mortality.

## Background

Patients with cancer have a 4- to 7-fold increased risk of suffering from venous thromboembolism (VTE) events, including deep venous thrombosis (DVT) and pulmonary embolism (PE) [1], a risk that is further augmented by cancer treatment [2, 3]. Development of VTE in the setting of cancer is associated with a significantly reduced life expectancy [3, 4].

There are several factors that have been implicated in increasing the risk of thrombosis in patients with cancer. The type of cancer is a strong predictor, with the strongest association being with malignant tumours of the pancreas, lung, stomach, adenocarcinomas of unknown primary, and of the ovary and brain; patients with locally advanced cancers and those with distant metastases may also be at increased risk of a VTE. Additionally, combination chemotherapy, including antiangiogenic agents, also reportedly heightens the risk [3, 4].

Analyses of the Kampala cancer registry data show a steady increase in the incidence of cancers in Uganda [5], with a corresponding rise in mortality [6]. Moreover, venous thrombosis has been consistently reported as a significant cause of mortality in cancer patients, especially in studies performed outside the sub-Saharan Africa [7]. The limited data on cancer associated thrombosis (CAT) in the sub-Saharan African have majorly been on case reports, clinical experience of radiological diagnosis and treatment, postmortem findings of hospital deaths and risk groupings of hospitalised patients [8]. Therefore, we undertook a retrospective study to describe the survival and clinical characteristics of patients with cancer-associated VTE in a tertiary oncology centre in Uganda.

## Methods

### Study design and setting

This was a retrospective study conducted at the Uganda Cancer Institute (UCI). UCI is the only tertiary cancer treatment facility in Uganda. Most patients with cancer in Uganda seek cancer care at the UCI. Occasionally, patients from the neighbouring countries, including the Democratic Republic of Congo, South Sudan, Kenya, Tanzania, Rwanda and Burundi, are also treated at the UCI.

The UCI provides both inpatient and outpatient services. Available options for cancer treatment at the UCI include chemotherapy, radiotherapy and surgery. Patients with CAT are also treated at the UCI. Available treatment options for thrombosis at the UCI include low-molecular weight heparin (LMWH), unfractionated heparin, warfarin and rivaroxaban.

Diagnosis of VTE at the UCI is undertaken using internationally recognised methodologies, primarily with vascular imaging [9]. Upon diagnosis, medications such as heparin and LMWH that require injections are given by the hospital nurses. Where possible, patients with CAT are treated as outpatients with oral medication. Prescriptions are normally recorded on the patient medical records/charts, which are securely maintained in a designated space at the UCI records offices.

### Eligibility criteria

Charts of eligible patients were selected using a homogenous purposive sampling method. Data were derived from charts of patients with radiologically confirmed DVT, PE or other VTE in association with a histologically confirmed cancer diagnosis at the UCI.

### Data collection

Data were manually derived using a standard data collection tool. Completed data collection tool was checked for completeness and accuracy by the principal investigator prior to acceptance for entry. Data were then coded, and entered into a computer using Epidata version 3.1 (Epidata association, Denmark) before exporting to STATA Version 14 (StataCorp, USA) for analysis. Study approvals and waivers of consent were obtained from the Uganda Cancer Institute Research Ethics Committee (UCIREC) and the Uganda National Council for Science and Technology (UNCST). All patient information was anonymised.

### Data analysis

Demographic and clinical characteristics were described using frequencies and percentages. One year overall survival (OS) rate and median survival were illustrated using the Kaplan–Meier curves. Survival was calculated from the date of initial diagnosis with thrombosis until the day of death, or until they were administratively censored. Cox proportional hazard model was used to evaluate the association between patient characteristics and OS at univariable and multivariable analyses starting with known factors associated with survival and then others. Patients who were lost to follow up were included in the analysis and were censored on the last recorded date of review at the UCI. Hazard ratios (HRs) and 95% confidence intervals were generated. Statistical significance was set at *p* < 0.05 (two-sided).

## Results

Charts of 111 patients who were seen from 2003 to 2019 met study eligibility and were included in the analysis. The mean (standard deviation (SD)) age of the study population was 52.4 (15.4) years. There were more female, 69 (62.2%), than male patients. Ninety four patients (84.7%) had a DVT, 12 (10.8%) had a PE and 3 (2.7%) had both DVT and PE. The most commonly represented cancer diagnoses were haematological cancers, 30 (27%), gynaecological cancers, 20 (18%) and prostate cancer, 17 (15.3%). The majority of patients, 87 (78.4%), presented with advanced cancer stage. Only four patients (3.6%) had early stage disease (haematologic), and 20 patients (18.0%) had no staging reported. The TNM system was used to stage solid tumours while Ann Arbor system was used to stage lymphomas; staging was not applicable to leukaemia. Thirty-eight (34.2%) patients had been exposed to chemotherapy within 6 months prior to the diagnosis of VTE, while 64 (67.7%) patients had not received any form of cancer treatment prior to the diagnosis of VTE. Table 1 shows the baseline characteristics of the study population.

### Treatment of VTE

The majority of patients, 100 (90.1%), were prescribed anticoagulation therapy. Although the intended duration of treatment was indicated on the prescription for all the patients, there was no information available to confirm that the patients completed the entire course. Therefore, data on duration of anticoagulation therapy were missing. Most patients, 72 (72%), were prescribed LMWH only therapy, while 22 (22%) were prescribed LMWH combined with warfarin. Other anticoagulation treatments were warfarin only therapy, 3 (3%), rivaroxaban, 2 (2%), and unfractionated Heparin, 1 (1%).

### Survival

**Figure 1.**
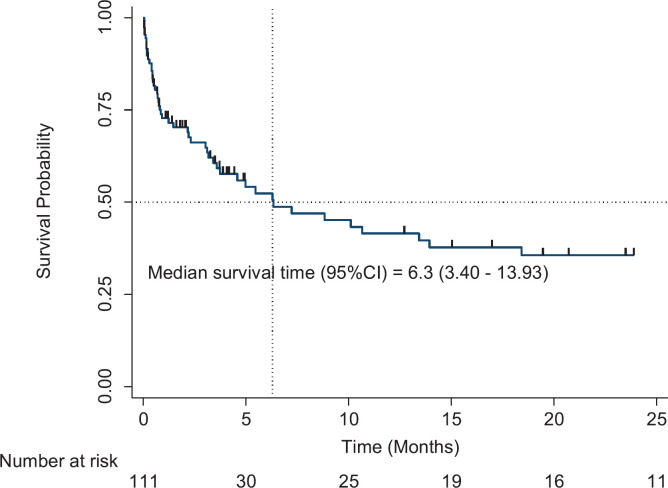
Overall survival of patients with CAT.

All patients enrolled in the study were followed up for survival analyses. The median OS for all patients with CAT was 6.3 months (95% CI, 3.40–13.93). The 1-year OS was 41.5% (95% CI, 29.7–52.9%), and the 2-year OS was 35.6% (95% CI, 24.1–47.4%) (Figure 1). Patients with the longest 1-year OS were those with KS, 87.5% (95% CI, 38.7–98.14), lung cancer, 50% (95% CI, 5.78–84.49%) and haematological cancers, 44.77% (95% CI, 23.6–63.9); others were gynaecological cancers, 42.4% (95% CI, 17.3–65.8), breast cancer, 38.1% (95% CI, 6.1–71.6), and prostate cancer, 37.3% (95% CI, 10.0–65.5). Factors that were analysed at both univariable and multivariable levels for predictors of mortality were age, sex, body mass index (BMI), type of cancer, stage of cancer, presence of comorbidities, and exposure to chemotherapy 6 months prior to VTE diagnosis. Patients with increased hazard of mortality had UGI malignancies (HR = 11.7, 95% CI, 30–59.13, *p* < 0.01), colorectal cancers (HR = 20, 95% CI, 1.95–205.9, *p* = 0.01) or breast cancer (HR = 8.2, 95% CI, 1.12–59.88, *p* = 0.04). Patients with a BMI of 25–29.9 kg/m^2^ (overweight) had a reduced hazard of mortality (HR = 0.1, 95% CI, 0.02–0.95, *p* = 0.04) (Table 2).

## Discussion

The median survival of patients with CAT in our study of 6.3 months was in line with most studies that have demonstrated that the presence of VTE in patients with cancer is an independent predictor of poor survival [10, 11], with almost all patients dying within 6 months of the diagnosis of thrombosis [12]. Additionally, a report of a large European database comparing the survival of patients with cancer and VTE with the survival of patients with cancer without VTE showed a markedly reduced 1-year survival in patients with VTE (12%) compared with those without VTE (36%).

Interestingly, some studies in New Zealand and the US have reported longer median survivals of 13.5 [13] and 16.7 [14] months, respectively. In the New Zealand study, survival was longest for haematological malignancy at 44.4 months, followed by prostate, bowel, breast, lung and pancreatic cancers at 29.4, 27.4, 15.5, 2.4 and 1.9 months, respectively [13]. Although our result showed a similarly high 1-year OS in patients with haematological malignan cies, the higher 1-year OS in patients with KS and lung cancers should be taken with caution due to the small sample size of both KS (8) and lung cancer (5). Notwithstanding, a recent study in Kenya reported excellent survival in patients with KS [15].

Nevertheless, the 1-year OS in our study of 41.5% was higher than reported in another population based study on a large European population, where the 1-year survival was only 12% [10]. Most patients in our study were prescribed LMWH (72%). It has been suggested that the OS in cancer patients with VTE treated using extended LMWH is longer than that reported from large registry and population studies in which speciﬁc patient information and therapeutic regimens were often unknown [13].

Patients with UGI and colorectal cancers had significantly higher hazard of mortality. Although our data do not provide reasons for this observation, we can only speculate that this might be due to the potential increased bleeding in patients with GI malignancies on anticoagulation therapy. Tetzlaff *et al* [16, 17] have reported that the presence of VTE in patients with advanced gastrooesophageal cancers leads to a poor OS. In a retrospective analysis of two large databases in California-US that included 68,142 patients with colorectal cancer, VTE was a significant predictor of death within 1 year of cancer diagnosis [18]. In a randomised trial on patients with colorectal cancer by Mandalà *et al* [19], patients with VTE had a significantly increased risk of mortality even after adjusting for age, disease site and treatment schedule. We have no immediate explanation for the increased hazard of mortality in patients with breast cancer.

The finding of a slightly reduced hazard of mortality in overweight patients is rather surprising, but is consistent with other studies. For example, in a single-institution cohort of 7,765 patients with gastric cancer, those who were overweight, mildly or moderately obese, had better OSs than patients with a normal BMI [20]. In another study to determine the effect of BMI on the risk of all-cause, cardiovascular, any cancer and breast cancer mortality in a cohort of older Caucasian women, patients with a BMI in the category of overweight had a lower risk of mortality [21]. Similarly, in a large study that included a cohort of 3,408 men and women diagnosed with colorectal cancer in the US, overweight patients (BMI: 25–28 and 28–<30) had lower mortality risks [22].

The younger age of presentation in our study population (mean age, 52 years) may be a reflection of the overall population age distribution in Uganda with the median age of 16.7 years [23]. The peak incidence of CAT in the UK patients occurred earlier among females than males. Furthermore, the finding of more females than males presenting with CAT is consistent with a European hospital study [24]. Although our data does not provide reason for this observation, some studies have attributed the higher representation of the female sex among patients with CAT to the possible use of hormone replacement therapy and hormonal contraceptives in some females of reproductive age [25].

The three most common cancers associated with thrombosis in our study were haematological, gynaecological and prostate cancers. A similar finding was reported in a study from Ibadan, Nigeria, where prostate cancer was the most common cancer associated with VTE [26]. However, the grouping of cancers into haematological and gynaecological types in our study may explain their over-representation. Studies in Europe and American have consistently reported VTE in patients with cancer of the pancreas, brain, liver, multiple myeloma, ovary and any form of advanced-stage cancer [12, 27].

We have added data on survival in patients with CAT in the sub-Saharan Africa. However, we acknowledge some major limitations including absense of the duration of anticoagulation for the patients. Additionally, haematological and gynaecological cancers were all conglomerate diagnoses. Distribution of these diagnoses would have provided a clearer picture of survival. It is also possible that our study had a uniquely selected patient population who were able to pay for the diagnostic workup for the VTE since compression/Doppler ultra sound scans and CT scans had to be paid for by the patients out-of-pocket. The small study sample size also suggests that our result should be taken with caution.

## Conclusion

In conclusion, our study suggests that there is a short OS of patients with CAT. Most of these patients present with advanced stage cancers and at a relatively younger age compared to those in developed countries. Patients with UGI, colorectal and breast cancers had increased hazards of mortality, whereas those who were overweight had a slightly reduced hazard of mortality. Further studies are suggested to evaluate the duration and outcome of anticoagulation therapies prospectively.

## Abbreviations

CAT, Cancer associated thrombosis; DVT, Deep venous thrombosis; LMWH, Low molecular weight heparin; PE, Pulmonary embolism; SD, Standard deviation; UCI, Uganda Cancer Institute; UCIREC, Uganda Cancer Institute Research and Ethics Committee; VTE, Venous thromboembolism

## Declarations

### Ethics approval and consent to participate

Waiver of consent and study approvals were obtained from the UCIREC (Reference number: 15-2017) and the study was registered at the UNCST (Reference number: HS 2412).

### Consent for publication

Not applicable.

### Availability of data and materials

All data generated or analysed during this study are included in this published article.

### Competing interests

All the authors have declared no conflicts of interest.

### Funding

This work was funded by the UCI. Any opinions, findings and conclusions expressed in this material are those of the author(s) and do not necessarily reflect those of the UCI. The funding source had no direct roles in the design of this protocol, data collection, analysis and interpretation, and manuscript writing.

### Authors' contributions

CDO: Designed the study, interpreted the data and wrote the manuscript. AO: Interpreted the data; HD; Interpreted the data; YM: Analysed and interpreted the data; JO: Interpreted the data. All authors read and approved the final manuscript.

### Previous presentation

None.

### Disclaimers

None.

## Figures and Tables

**Table 1.** Baseline characteristics.

Factor	Data
Age, mean (SD) years	52.4 (15.4)
Female sex, *n* (%)	69 (62.2)
**Type of VTE, *n* (%)**	
DVT	94 (84.7)
PE	12 (10.8)
DVT and PE	03 (2.7)
Other sites of thrombosis	02 (1.8)
**BMI (kg/m^2^), *n* (%)**	
Underweight (<18.5)	11 (9.9)
Normal weight (18.5–24.9)	14 (12.6)
Overweight (25–29.9)	06 (5.4)
Obese (>30)	05 (4.5)
Unknown	75 (67.6)
**Type of malignancy, *n* (%)**	
Haematological	30 (27.0)
Gynaecological	20 (18.0)
Prostate	17 (15.3)
Breast	08 (7.2)
Kaposi Sarcoma (KS)	08 (7.2)
Upper gastrointestinal (UGI)	07 (6.3)
Lung	06 (5.4)
Colorectal	05 (4.5)
Dual malignancy (UGI + prostate )	01 (1.0)
Other	09 (8.1)
**Cancer stage, *n* (%)**	
Stage I	01 (0.9)
Stage II	09 (8.1)
Stage III	31 (28.0)
Stage IV	50 (45)
Not applicable	20 (18.0)
**Cancer treatment 6 months prior to VTE diagnosis, *n* (%)**	
Chemotherapy	38 (34.2)
Radiotherapy	03 (2.7)
Surgery	01 (0.9)
Two or more modalities	05 (4.5)
None	64 (67.7)

**Table 2.** Predictors of mortality.

Variable	CHR^a^ (95% CI)	*p*-value	AHR^a^ (95% CI)	*p*-value
Age (years)	1 (0.98–1.02)	0.96	1 (0.97–1.02)	0.84
**Sex**				
Male	1		1	0.22
Female	1.2 (0.65 - 2.06)	0.61	0.6 (0.26–1.35)	
**BMI group**				
Underweight (<18.5)	1		1	
weight (18.5–24.9)	0.8 (0.24–2.58)	0.69	0.6 (0.18–2.2)	0.46
Overweight (25–29.9)	0.3 (0.06–1.91)	0.22	0.1 (0.02–0.95)	0.04
Obese (30 and above)	1.5 (0.37–5.99)	0.58	1.2 (0.26–5.93)	0.78
Unknown	0.6 (0.22–1.84)	0.41	0.6 (0.18–1.99)	0.40
**Cancer stage**				
Early stage	1		1	
Late stage	0.5 (0.22–0.92)	0.03	0.6 (0.10–3.41)	0.55
Missing	0.4 (0.14–1.01)	0.05	2.3 (0.37–13.9)	0.38
**Comorbidities**				
No	1		1	
Yes cardiovascular	1 (0.42–2.21)	0.92	0.7 (0.26–1.83)	0.46
Yes other	1.2 (0.66–2.21)	0.54	1.9 (0.88–4.02)	0.10
**Gynaecological cancer**				
No	1		1	
Yes	1.3 (0.66–2.51)	0.46	3 (0.65–13.42)	0.16
**Colorectal cancer**				
No	1		1	
Yes	2.7 (0.64–11.48)	0.18	20 (1.95–205.9)	0.01
**UGI cancer**				
No	1		1	<0.01
Yes	3.1 (1.22–7.94)	0.02	11.7 (2.3–59.13)	
**Lung cancer**				
No	1		1	0.92
Yes	0.6 (0.14–2.34)	0.43	0.9 (0.12–6.84)	
**Prostate cancer**				
No	1		1	0.22
Yes	0.9 (0.4–1.82)	0.69	2.6 (0.55–12.45)	
**Haematological cancer**				
No	1		1	0.58
Yes	0.9 (0.47–1.6)	0.64	1.5 (0.35–6.61)	
**Breast cancer**				
No	1		1	0.04
Yes	1.2 (0.47–2.98)	0.73	8.2 (1.12–59.88)	
**KS**				
No	1		1	0.12
Yes	0.2 (0.03–1.45)	0.11	0.1 (0.01–1.71)	
**Chemotherapy before VTE diagnosis**				
No	1		1	0.42
Yes	0.8 (0.45–1.43)	0.45	0.7 (0.32–1.62)	

a Note: AHR, Adjusted hazard ratio; CHR, Crude hazard ratio
